# The Largest Statue in the World

**Published:** 1888-02

**Authors:** 


### THE LARGEST STATUE IN THE WORLD.

Near the small town of Bamian, in Afghanistan, at the foot of the Hindoo-Koosh chain of mountains, several colossal statues were discovered about a year ago, which, in point of size, excel any representation of the human form ever carved by the hand of man.

The valley in which Bamian is situated is bordered by precipitous cliffs of a hard conglomerate rock ; and in the sides of one of the cliffs five immense statues have been cut out of the solid rock, the largest, of which, is no less than a hundred and seventy-three feet in height. When it is remembered that the Statue of Liberty in New York harbor is but a hundred and thirty-seven feet high, the immense proportions of this remarkable work of antiquity will be better appreciated. The interior of this niche was covered with paintings of human figures, some of which are still well preserved.

Rude galleries and staircases are cut in these figures, by which access can be gained to the heads, the same as in our modern colossal statues. Some time after the completion of the statue, its draperies were formed by masses of stucco moulded into their proper shape and fastened to it. Numerous holes, still visible, are said to have been made for the purpose of holding the stucco in place.

The general appearance of these statues indicates that they were the work of the Buddhist monks, who were very numerous in this region about the time of the Christian era. The largest one, at least, was doubtless meant to be a representation of Buddha. They were probably made about nineteen hundred years ago. Alexander the Great, passed through this valley in the fourth century before Christ, but the historians of his expedition make no mention of them. A Chinese pilgrim, Huen Tsang, visited Bamian about 630 A. D., and gave the first description of which we have any knowledge ; but even then the statues were very old, and the handful of monks remaining would have been utterly incapable of performing such an immense work.

It is to the modern English and Russian explorers that we are indebted for the rediscovery of these remarkable monuments, which are certainly worthy of being ranked among the ancient wonders of the world.

